# Comparative Genome Analysis Reveals Phylogenetic Identity of Bacillus velezensis HNA3 and Genomic Insights into Its Plant Growth Promotion and Biocontrol Effects

**DOI:** 10.1128/spectrum.02169-21

**Published:** 2022-02-02

**Authors:** Doaa S. Zaid, Shuyun Cai, Chang Hu, Ziqi Li, Youguo Li

**Affiliations:** a State Key Laboratory of Agricultural Microbiology, Huazhong Agricultural University, Wuhan, People’s Republic of China; b Desert Research Center, Arab, Republic of Egypt; University of Minnesota

**Keywords:** genome sequencing, comparative analysis, plant growth-promoting rhizobacteria, *Bacillus velezensis* HNA3, gene cluster, carbohydrate active enzymes

## Abstract

Bacillus velezensis HNA3, a potential plant growth promoter and biocontrol rhizobacterium, was isolated from plant rhizosphere soils in our previous work. Here, we sequenced the entire genome of the HNA3 strain and performed a comparative genome analysis. We found that HNA3 has a 3,929-kb chromosome with 46.5% GC content and 4,080 CDSs. We reclassified HNA3 as a Bacillus velezensis strain by core genome analysis between HNA3 and 74 previously defined *Bacillus* strains in the evolutionary tree. A comparative genomic analysis among Bacillus velezensis HNA3, Bacillus velezensis FZB42, Bacillus amyloliquefaciens DSM7, and Bacillus subtilis 168 showed that only HNA3 has one predicated secretory protein feruloyl esterase that catalyzes the hydrolysis of plant cell wall polysaccharides. The analysis of gene clusters revealed that whole biosynthetic gene clusters type Lanthipeptide was exclusively identified in HNA3 and might lead to the synthesis of new bioactive compounds. Twelve gene clusters were detected in HNA3 responsible for the synthesis of 14 secondary metabolites including Bacillaene, Fengycin, Bacillomycin D, Surfactin, Plipastatin, Mycosubtilin, Paenilarvins, Macrolactin, Difficidin, Amylocyclicin, Bacilysin, Iturin, Bacillibactin, Paenibactin, and others. HNA3 has 77 genes encoding for possible antifungal and antibacterial secreting carbohydrate active enzymes. It also contains genes involved in plant growth promotion, such as 11 putative indole acetic acid (IAA)-producing genes, spermidine and polyamine synthase genes, volatile compound producing genes, and multiple biofilm related genes. HNA3 also has 19 phosphatase genes involved in phosphorus solubilization. Our results provide insights into the genetic characteristics responsible for the bioactivities and potential application of HNA3 as plant growth-promoting strain in ecological agriculture.

**IMPORTANCE** This study is the primary initiative to identify Bacillus velezensis HNA3 whole genome sequence and reveal its genomic properties as an effective biocontrol agent against plant pathogens and a plant growth stimulator. HNA3 genetic profile can be used as a reference for future studies that can be applied as a highly effective biofertilizer and biofungicide inoculum to improve agriculture productivity. HNA3 reclassified in the phylogenetic tree which may be helpful for highly effective strain engineering and taxonomy. The genetic comparison among HNA3 and closely similar species B. velezensis FZB42, *B. amyloliquefaciens* DSM7, and B. subtilis 168 demonstrates some distinctive genetic properties of HNA3 and provides a basis for the genetic diversity of the *Bacillus* genus, which allows developing more effective eco-friendly resources for agriculture and separation of Bacillus velezensis as distinct species in the phylogenetic tree.

## INTRODUCTION

The plant rhizosphere, a small zone of soil around a developing plant’s root system, is a hot spot for microbial activity (1) where a diverse range of microbial species inhabits the rhizosphere region (2). Plant growth-promoting bacteria are free-living bacteria that flourish in this region and exuberantly colonize roots to enhance plant growth (3). Genus *Bacillus* is one of the most common and genetically varied groups of easily cultivable PGPRs (4). *Bacilli* have a great interest in agro-systems according to their great potential to enhance agricultural productivity and control crops diseases because of their active rhizosphere colonization and PGP properties (5).Upon these characteristics, some *Bacillus* strains can be used as environmental-friendly biofertilizer and biofungicide inoculants in agriculture and horticulture.(6). Recently, great attention has been paid to various strains of Bacillus velezensis which are novel species of *Bacillus* (7) that are deemed as vital PGPR. B. velezensis was initially described by Wang et al. in 2008 as a heterotypic synonym of *B. amyloliquefaciens*. They produce a wide variety of bioactive secondary metabolites such as antibiotics. These secondary metabolites primarily comprise non-ribosomal peptides and polyketides synthesized by non-ribosomal peptide synthetases (NRPS) and polyketide synthases (PKS), respectively (8). More specifically, B. velezensis exhibits a high genetic capacity for synthesizing cyclic lipopeptides (i.e., Surfactin, Bacillomycin D, Fengycin, and Bacillibactin) and polyketides (i.e., Macrolactin, Bacillaene, and Difficidin) (9). These antibiotics enhance crop stress tolerance and suppress pathogenic fungi, affecting the health of plants (10). Growth promotion processes and bacterial communication operation between B. velezensis strains and plants are still under exploration (11). Growth promotion in plants is believed to be the consequence of a complicated set of mechanisms that impact both plant development and nutrition (12) both directly and indirectly. B. velezensis has a beneficial impact on plant development. It stimulates plant development directly by facilitating nutrient absorption such as solubilizing of phosphate (13)and nitrogen fixation (14), producing or regulating plant hormones such as IAA (15), producing active volatile compound that enhances plant induced systemic resistance (ISR) (16). It is also distinguished by the release of carbohydrate-active enzymes (CAZymes) (17), which have the capacity to breakdown the most complicated substrates such as complex carbohydrates and use it as a simple source of nourishment for the synthesis of vital compounds (18) besides its crucial role in their capacity to hydrolyze the cell wall of plant pathogenic microorganisms (19). There is a wide range of indirect processes whereas B. velezensis PGPR influences plant development and disease prevention and suppression (20). B. velezensis species are favored in farm systems due to their capability of forming endospores that can tolerate drought and heat. In addition, it can be formulated into stable long-lived dry powders (21). Genetic studies and genetic comparison are extremely helpful for the understanding of the biological properties of PGPR. Numerous studies have reported the significant dual activity of newly isolated B. velezensis species. It is worth mentioning that only 47 strains within B. velezensis were performed for whole genome sequencing and 20 of them have been totally assembled (22).

Lately, several Bacillus methylotrophicus, *B. amyloliquefaciens subsp. plantarum*, and *Bacillus oryzicola*were reclassified as *B. velezensis* based on comparative genomics and DNA–DNA relatedness calculations (23). Generally, it is very difficult to identify *B. amyloliquefaciens*, B. subtilis, and *B. velezensis* based on 16S rRNA gene sequence similarity analysis, morphological observation, and physiological and chemical reactions due to high conserved nature of the gene (24). Development of bioinformatics tools and techniques were utilized to identify and differentiate new species in *Bacillus* taxa (25).The genome comparison between these closely related species reveals the evolutionary genetic change that is suggested to happen according to several factors one of which microorganism associated ecological environment (26).

*B. velezensis* HNA3 (referred to as HNA3 hereafter) is a rod-shaped and Gram-positive bacterium arranged in pairs or chains and produces endospores when cultivated on an LB medium. HNA3 colony is irregular and opaque with an off-white color, and was previously isolated from the rhizosphere soil by our group. Xu et al. (2013) reported the morphological characteristics of HNA3, separated the lipopeptide compounds with a broad spectrum of antifungal bioactivities, proposed the optimum cultivation conditions of HNA3, and classified it as *B. amyloliquefaciens* based on morphological observations and 16S rRNA sequence analysis (27).

## THE OBJECTIVE OF THIS STUDY

This manuscript aims to identify genome sequence of B. velezensis HNA3, and classify strain HNA3 based on whole-genome and phylogenetic analysis. We also will perform comparative genomic analysis among genome sequences of reference strains B. velezensis FZB42, B. amyloliquefaciens DSM7, and B. subtilis 168 with HNA3, in addition to evaluating how genes and gene clusters encoding potential secondary metabolites of B. velezensis HNA3 contribute to plant growth-promoting ability and biocontrol activity. This research lays the groundwork for future investigations of target genes and functions, as well as genetic engineering of HNA3 to optimize agricultural and industrial applicability.

## RESULTS

### Genomic features of HNA3.

**FIG 1:**
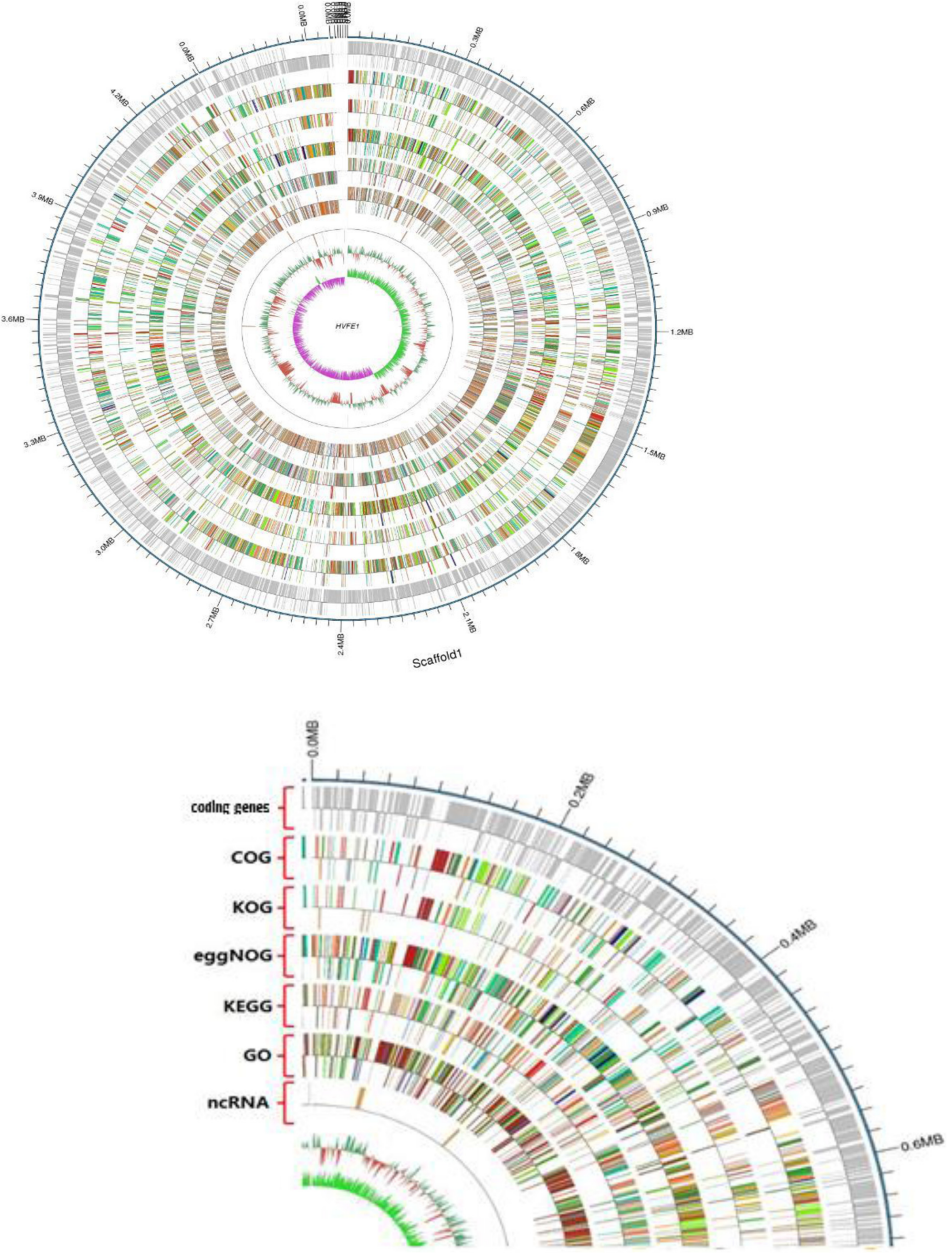
HNA3-Chromosome genome-wide dynamic map. The outer edge circle is the genomic sequence position coordinating from outside to inside (coding gene, annotation results for gene-functions; COG [KOG], KEGG, GO, and ncRNA). The GC content was calculated by window (chromosome length/1,000) bp, step size (chromosome length/1,000) bp. The inward red part indicates that the GC content of this region is lower than the average of the whole genome; the outward green part is converse, and a higher peak value indicates a greater difference from the average GC content. The specific algorithm is GC/G+C. The inward pink portion indicates that the content of the region G is lower than that of C, and the outward light green portion is opposite.

The Bacillus velezensis HNA3 genome was sequenced using Single-Molecule Real-Time (SMRT) technology, yielding 4,161 MB of raw data. Following quality control, 3,965 MB of data was collected and assembled, giving nine integrated contigs. The main characteristics of the HNA3 genome are summarized in Fig. 1. It has one circular chromosome with 3,929,648 bp, an average of 46.5% guanine-cytosine percentage in DNA (GC content), and one plasmid (3,321 bp, GC content 2.11%); it also possesses a total of 4,202 genes (4,080 a protein-coding sequences CDSs [a coding sequence is a regions of DNA or RNA that that corresponds with the sequence of amino acids in a protein] and 122 RNA genes).

### Phylogenetic analysis of HNA3 and average nucleotide identity.

**FIG 2:**
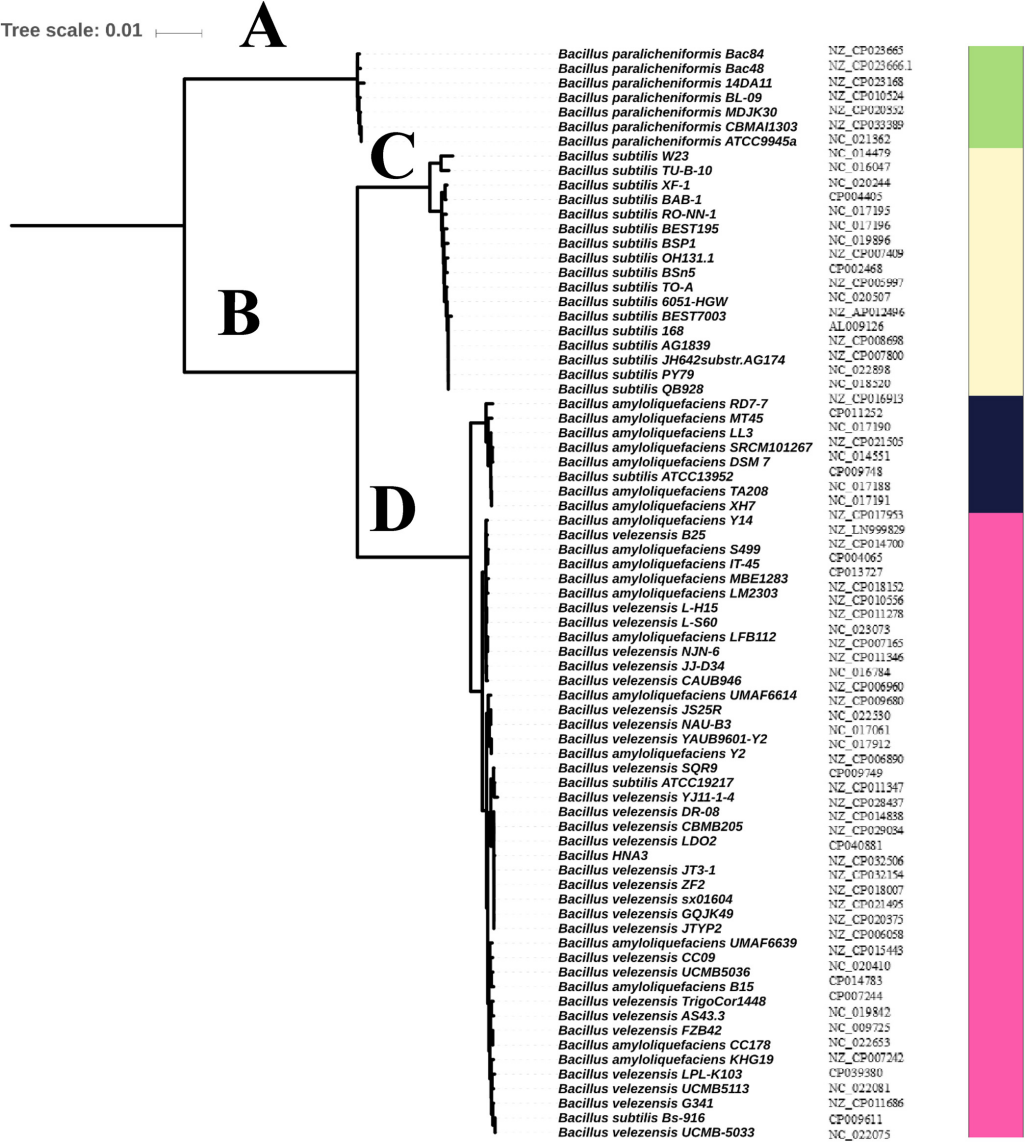
Maximum likelihood tree for 74 *Bacillus* strains. Different colors indicate different tree clades. Green color indicates the members of first clade (A). Yellow color indicates the first branch of the second clade (C). Blue and pink indicate the second branch of the second clade (D).

To evaluate the phylogenetic position of HNA3, based on the alignment of nucleotide sequences for the 293 single-copy core genes, we created a maximum likelihood phylogenetic tree that were preserved in a single copy in the genomes of all strains presented in the phylogenetic tree (Fig. 2). Our collection consists of 21 B. subtilis strains, 18 B. amyloliquefaciens strains, 28 B. velezensis strains, and seven B. paralicheniformis strains (designed as an outgroup).

The phylogenetic tree showed that most strains were clustered into two separate phylogenetic clades (A, B) with a common ancestor. The first clade (A) only included all strains of B. paralicheniformis, while the second clade (B) comprised all strains of B. subtilis strains, B. amyloliquefaciens, and B. velezensis. The second clade could be further divided into two branches (C, D), with the first branch (C) including all strains of B. subtilis, and the second branch (D) containing all strains of B. amyloliquefaciens and B. velezensis with the same ancestor. Also, there were great similarities between these two species, but recently they have been separated according to the genetic marker differentiation. HNA3 shared the same region with other B. velezensis strains in the phylogenetic tree. As shown in Fig. S1, the average nucleotide identity (ANI) of the strains in the first clade (A) was > 78.7%, while that of all strains in the first branch from the second clade (C) was > 81.0%, and that of the strains in the second branch from the second clade (D) was > 98.6%, indicating high similarities between HNA3 and the species in the second branch of the second clade (D).

### Phylogenetic position of HNA3 among the 15 closely related strains.

**FIG 3:**
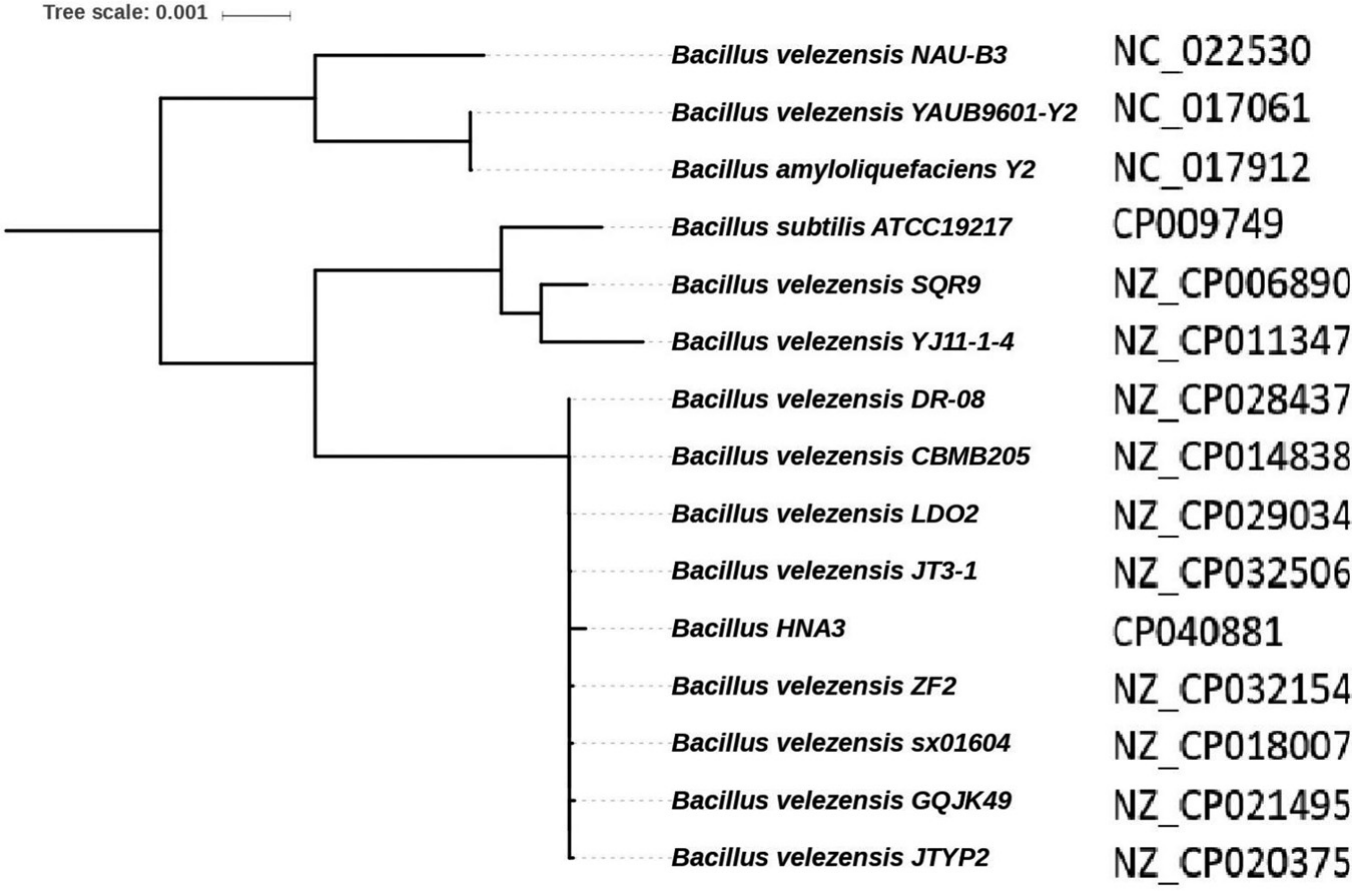
Maximum likelihood tree for HNA3 and 15 closely related strains. The inner tree node is bootstrap (100 replicates) values; values above 80 are provided.

A maximum-likelihood tree of 293 single-copy genes was constructed, which was shared by15 strains closely associated with HNA3 (Fig. 3). These 15 strains had similar genetic characteristics and genome sizes (3.7 to 3.9 Mb), with the number of CDSs ranging from 3,656 to 4,080.

The phylogenetic tree of the15 strains was segregated into three different sub-lineages that support the high bootstrap values. Sub-lineage І included *B. velezensis* NAU-B3 and *B. velezensis* YAUB9601-Y2; sub-lineage II comprised B. subtilis ATCC19217, *B. velezensis* SQR9, and *B. velezensis* YJ11-1-4; sub-lineage III consisted of HNA3 and eight other strains, including *B. velezensis* DR-08, CBMB205, LDO2, JT3-1, ZF2, sx01604, GQJK49, and JTYP2. The ANI results showed that HNA3 had about 99.8% homology to *B. velezensis* JT3, LDO2, and CBMB205.

To reveal the differences at the nucleotide level, the genome of HNA3 was used as a reference to align the fully sequenced genomes of other 15 *B. velezensis* strains using the BLAST Ring Image Generator (BRIG). The results revealed that the HNA3 genome sequence has more than 98% similarity to the genome sequences of these 15 strains (Fig. S2). Notably, the HNA3 genome exhibited more than 99.1% similarities to those of *B. velezensis* JT3, LDO2, and CBMB205.

**FIG 4:**
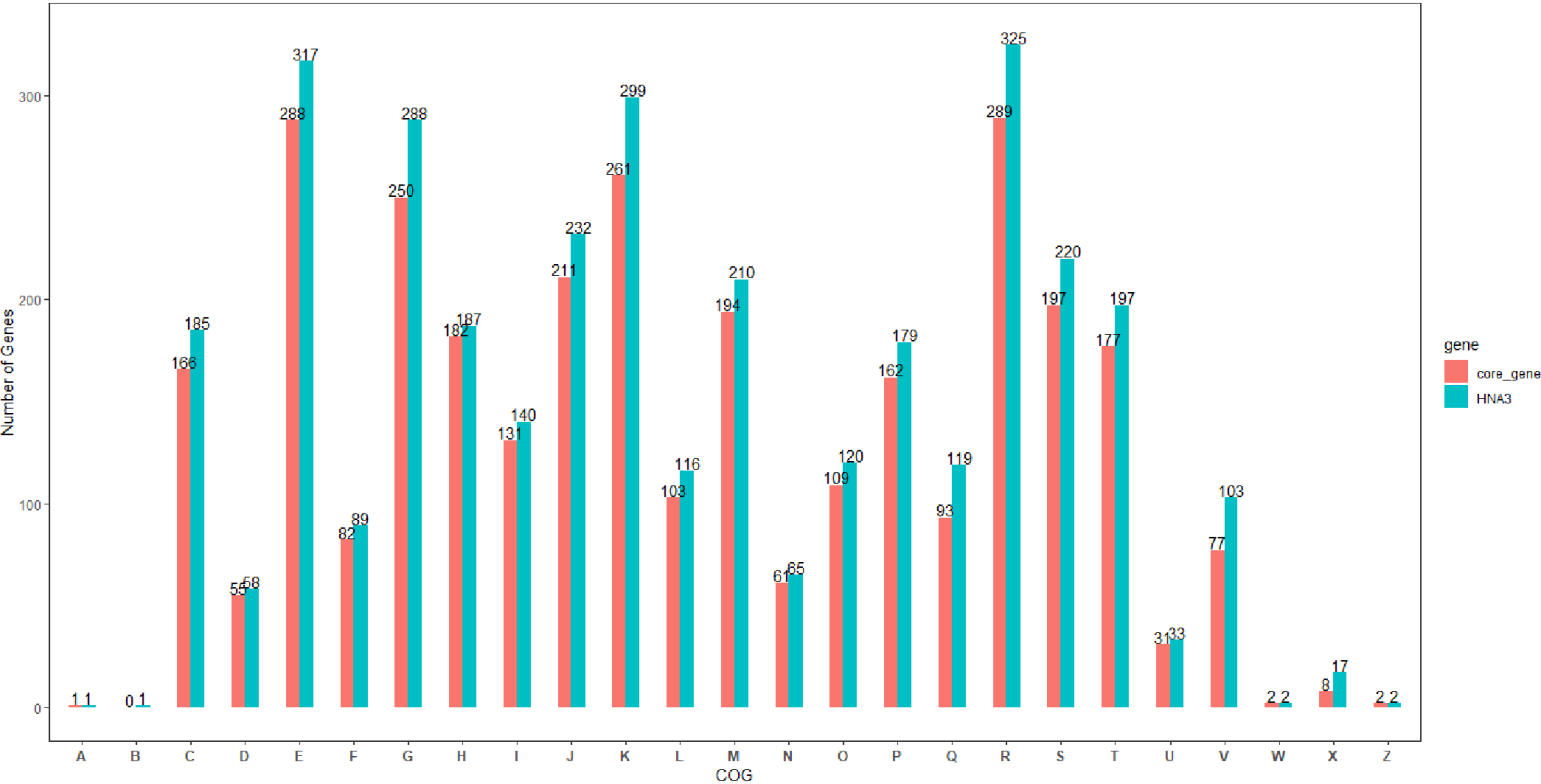
Functional Comparison of COG categories among HNA3 CDSs and core genome of 15 *Bacillus* strains closely related with HNA3. The comparison based on 26 COGs categories provided by the COG database. Blue column for HNA3 while red column represents core genome of 15 closely related strains. Number above each column indicates to the number of genes relative to each COG functional category. COGs categories are : RNA processing and modification (A), chromatin structure and dynamics (B), energy production and conversion (C), cell cycle control, cell division, chromosome partitioning (D), amino acid transport and metabolism (E), nucleotide transport and metabolism (F), carbohydrate transport and metabolism (G), coenzyme transport and metabolism (H), lipid transport and metabolism (I), translation, ribosomal structure and biogenesis (J), transcription (K), replication, recombination and repair (L), cell wall, membrane, envelope biogenesis (M), cell motility (N), posttranslational modification, protein turnover, chaperones (O), inorganic transport and metabolism (P), secondary metabolites biosynthesis, transport and catabolism (Q), general function prediction only (R), function unknown (S), signal transduction mechanisms (T), intracellular trafficking, secretion and vesicular transport (U), defense mechanisms (V), extracellular structures (W), mobilome: prophages, transposons (X), nuclear structure(Y), cytoskeleton (Z).

The cluster of orthologous group (COG) assignment was performed to determine the category of the gene function of HNA3 and the core genome of the 15 very closely related strains to evaluate the genetic diversity (Fig. 4). As shown in the COG assignment, 317 HNA3 genes were allocated to amino acid transport and metabolism; 299 were assigned to transcription; 288 were annotated for carbohydrate transport and metabolism functions; and 199 were assigned for secondary metabolite biosynthesis, transport, and catabolism.

### Comparison of genetic characteristics between B. velezensis HNA3 and Bacillus amyloliquefaciens DSM7, B. velezensis FZB42, and Bacillus subtilis 168.

Genetic characteristics of HNA3 with three other reference *Bacillus* strains, including B. amyloliquefaciens DSM7 (non-plant associated strain), B. velezensis FZB42 (plant-associated rhizobacteria), and B. subtilis 168 from the different biological environment was further analyzed. The general features of these strains are illustrated in Table 1.

**TABLE 1:** Genomic comparison between *Bacillus velezensis* HNA3, *Bacillus velezensis* FZB42, *Bacillus amyloliquefaciens* DSM7, and *Bacillus subtilis* 168

General genome characteristics	*B*.HNA3	*B*.FZb42	*B*.DSM7	*B*.168
	Plant-associated rhizobacteria	Plant-associated rhizobacteria	Non-plant-associated strain	Domestic
NCBI accession	CP040881	NC_009725	NC_014551.1	AL009126.3
Size (base pairs)	3929648	3918596	3980199	4215606
Genomic Island	7	8	9	4
Prophage	14	15	11	9
Pseudo genes	236	202	234	201
rRNA	27	29	30	30
Transporter	86	86	85	102
ncRNA	249	267	267	278
rRNA	27	30	30	30
tRNA	86	88	94	87

**FIG 5:**
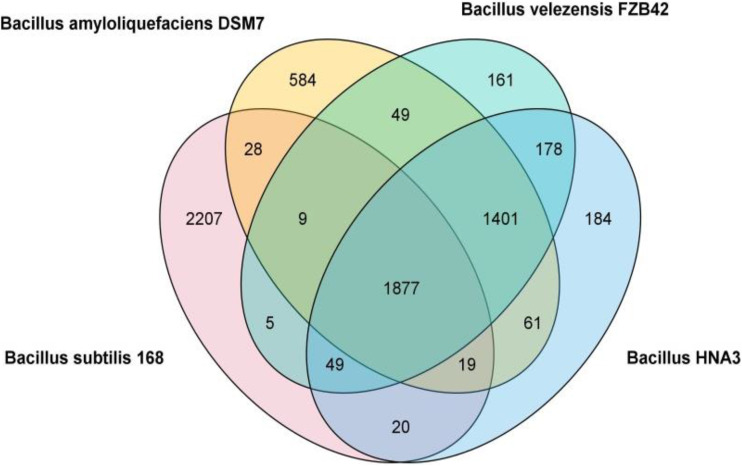
Venn diagram of Bacillus velezensis HNA3, Bacillus velezensis FZB42, Bacillus amyloliquefaciens DSM7, and Bacillus subtilis 168. The numbers of CDS between subsets of genomes are shown.

A comparative analysis among the CDSs of the four *Bacillus* strains showed that 1,877 genes were shared by all four selected *Bacillus* strains (Fig. 5). A total of 1,965 genes were common between HNA3 and B. subtilis 168. HNA3 shared 3,505 genes with B. velezensis FZB42 while shared 3,358 genes with B. amyloliquefaciens DSM7. On the other hand, there were 184 single-copy genes assigned for the HNA3 strain only. HNA3 had 431 and 284 unique genes relative to B. amyloliquefaciens DSM7 and B. velezensis FZB42, respectively.

Artemis comparison tool (Web ACT) was used to detect the preserved and unique regions of the genome, which would reveal different biological behavior so certain adaptations. In the comparison among HNA3, FBZ42, DSM7, and 168 (Fig. S3) the genomes of HNA3 and FBZ42 showed many conserved sequences and genetic regions, while DSM7 and 168 had fewer conserved sequences and regions, possibly because HNA3 and FBZ42 were derived from the same source (plant-associated strains) and there was a high degree of similarity between them.

The comparison of predicted proteins in the HNA3 genome and the three additional *Bacillus* strains (DSM7, FZB42, and 168) for CAZymes, illustrated in Table 2, showed that HNA3 has 39 glycoside hydrolases enzymes (GHs), 36 glycosyltransferases enzymes (GTs), three polysaccharide lyases enzymes (PLs), nine carbohydrate esterase (CEs), and one auxiliary activities (AAs), as well as 27 carbohydrate-binding modules proteins (CBMs), which play an important role in enhancing enzyme-substrate binding. Only three predicted proteins of carbohydrate active enzymes were identified in the genome of HNA3 and 168: α,α-trehalase, 4-α-d-glucan glucono hydrolase, and cyclohepta glucanase (Reference Accession: QJC43453, QJC40906, and QJC43458 respectively); α,α-trehalase enzyme catalyzes the hydrolysis of the α-glucosidic *O*-linkage of α,α-trehalose and releases α- and β-d-glucose(28). 4-α-d-glucan glucono hydrolase enzyme acts on starch, glycogen, and oligosaccharides in a random manner (29). Cyclohepta glucanase enzyme binds to cyclic maltodextrin complex creating the ring-opening to make it easy to be hydrolysis (30). Predicated protein, lytic chitin monooxygenase enzyme (QJC42116), has been found in the genome of HNA3, FZB42, and DSM7, but not in 168.

**TABLE 2:** Numerical comparison of predicated carbohydrate active enzymes families among HNA3 and other three *Bacillus* strains (FZB42, DSM7, and 168)

*Bacillus* strains	Glycoside hydrolase enzymes	Glycosyl transferase enzymes	Polysaccharide lyase enzymes	Carbohydrate esterase enzymes	Auxiliary activity enzymes	Carbohydrate-binding module enzymes
HNA3	39	36	3	9	1	27
FBZ42	38	36	3	8	1	21
DSM7	42	37	3	8	0	24
168	51	34	7	11	0	37

One predicated protein feruloyl esterase enzyme (QJC42740) has been found exclusively in the genome of HNA3 that catalyzes the hydrolysis of feruloyl group from arabinose (plant gums). All ferulate esterases produced by microbes are secreted into the culture medium. They are frequently referred to as hemicellulase supplementary enzymes because they help xylanases and pectinases in the breakdown of plant cell wall hemicellulose (31).

**FIG 6:**
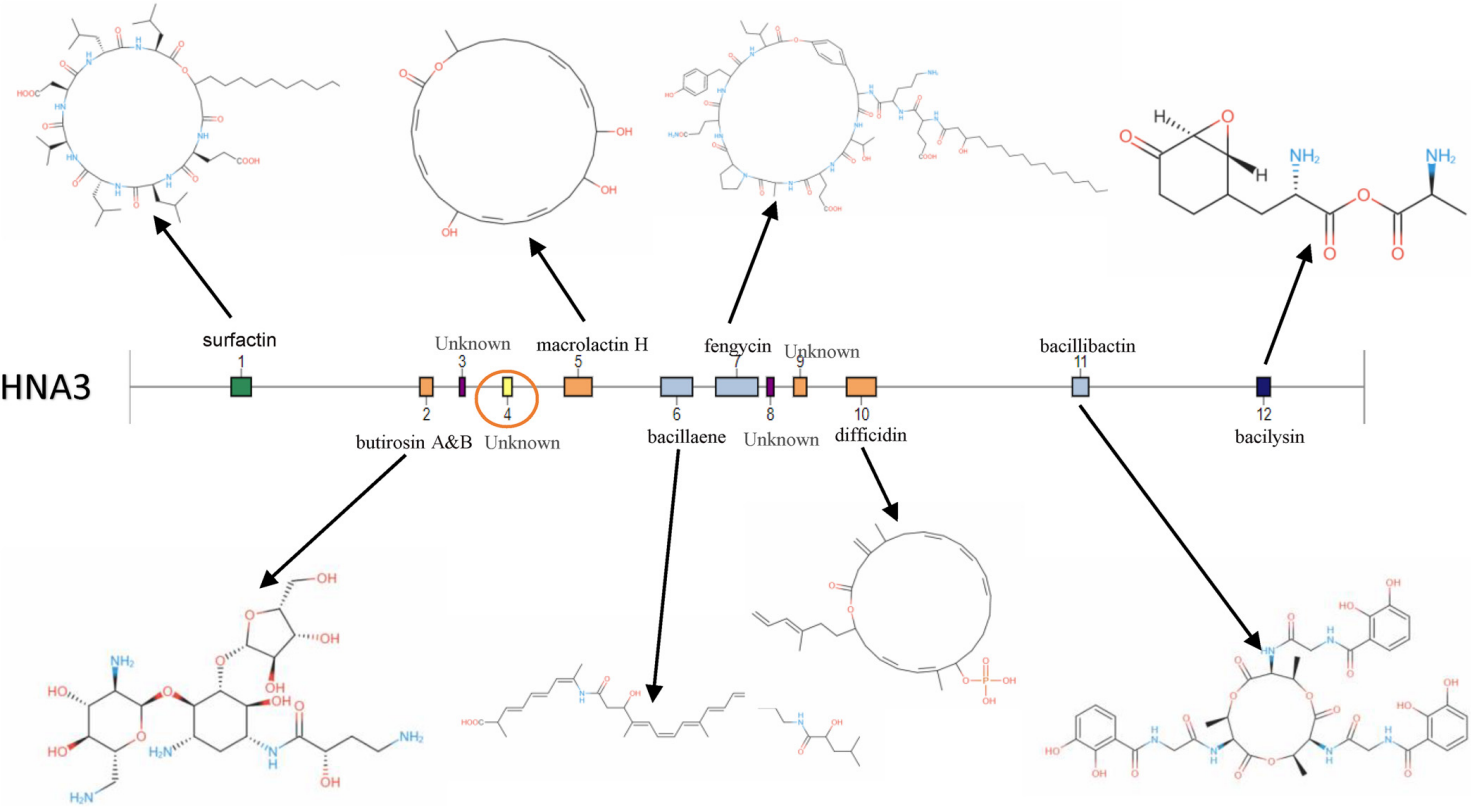
Gene cluster identified in HNA3 genome using antiSMASH software version 5.1.0.

Through genome mining, using antiSMASH software version 5.1.0, 12 secondary metabolite clusters have been identified in the genome of *B. velezensis* HNA3, three encoding NRPS, three Trans ATPKS, two Terpene, one PKS, one Lanthipeptide, one T3PKS, and one other KS. Clusters responsible for the synthesis of Surfactin, Macrolactin, Bacillaene, Fengycins, Difficidin, Bacillibactin, and Bacilysin have been detected in the B. velezensis HNA3 genome (Fig. 6).

Comparison of gene clusters among HNA3 and the three additional *Bacillus* strains (DSM7, FZB42, and 168) illustrated that there are six clusters (1, 3, 6–8, 11) that are conserved by the four strains of *B. velezensis* HNA3, *B. velezensis* FZB42, *B. amyloliquefaciens* DSM7, and B. subtilis 168. Two clusters (2, 9) are conserved by the three strain of *B. velezensis* HNA3, *B. velezensis* FZB42, and *B. amyloliquefaciens.* Two clusters (5 and10) are identified only in the two strains of *B. velezensis* (HNA3 and FZB42) (Table 3).

**TABLE 3:** Comparison analysis of gene cluster type, location, compound, and size among Bacillus velezensis HNA3 and Bacillus velezensis FZB42, Bacillus amyloliquefaciens DSM7 and Bacillus subtilis 168

HNA3		Gene cluster location				Presence (+) or absence (-)		
Cluster no.	Type	From	To	Compound	Size (nt)	FZB42	DSM7	168
1	NRPS	323,406	387,383	Surfactin	63,978	+	+	+
2	PKS-like	924,042	965,286	Butirosin A/Butirosin B	41,245	+	+	-
3	Terpene	1,050,165	1,067,573	Unknown	17,409	+	+	+
4	Lanthipeptide	1,188,553	1,217,440	Unknown	28,888	-	-	-
5	TransAT-PKS	1,384,047	1,471,873	Macrolactin	87,827	+	-	-
6	TransAT-PKS, T3PKS, NRPS	1,691,394	1,791,959	Bacillaene	100,566	+	+	+
7	NRPS, TransAT-PKS, Betalactone	1,865,682	1,999,991	Fengycin	134,310	+	+	+
8	Terpene	2,028,629	2,050,512	Unknown	21,884	+	+	+
9	T3PKS	2,113,830	2,154,930	Unknown	41,101	+	+	-
10	TransAT-PKS	2,282,301	2,376,092	Difficidin	93,792	+	-	-
11	NRPS, RiPP-like	3,000,753	3,052,541	Bacillibactin	51,789	+	+	+
12	Other	3,588,840	3,630,258	Bacilysin	41,419	+	-	+

**FIG 7:**
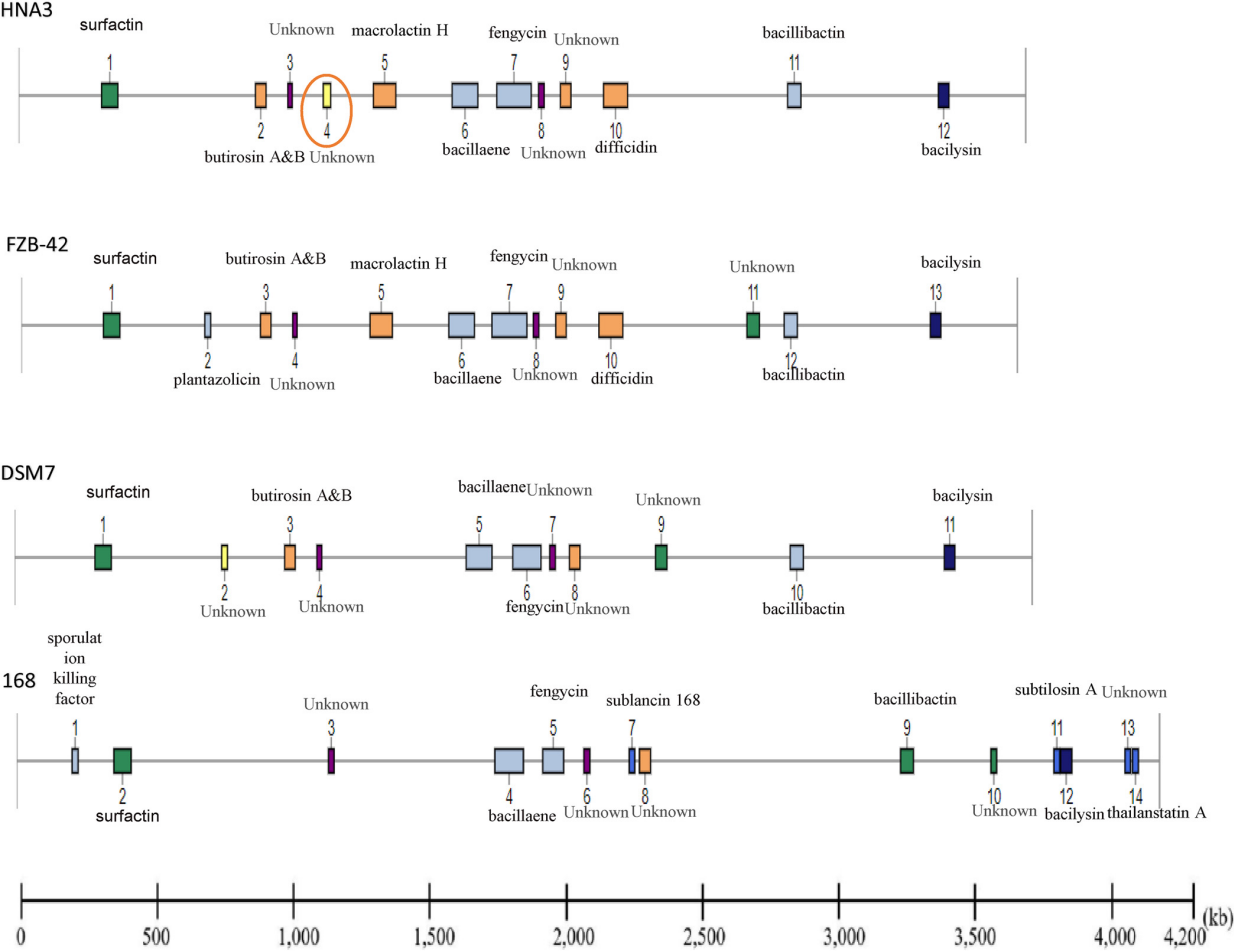
Comparison of gene clusters among Bacillus velezensis HNA3, Bacillus velezensis FZB42, Bacillus amyloliquefaciens DSM7, and Bacillus subtilis 168. Cluster No.4 in HNA3 genome not presented in other three genomes.

Another cluster (4) encoding Lanthipeptide was identified exclusively in the *B. velezensis* HNA3 genome, based on a comparative genomics study; this might lead to the production of novel bioactive compounds. In the same way, we found three clusters (3, 8, 9) encoding terpene and T3PKs with no previously reported description due to the limited similarity of the compounds deposited in the minimum information about a biosynthetic gene cluster (MIBiG) database (Fig. 7).

Although several gene clusters were shared by the four strains (HNA3, FZB42, DSM7, and 168), the gene structure is different in them. For example, the Surfactin cluster in HNA3 lacks *ycxC* and *ycxD* genes. The same cluster in DMS7 lacks *ycxC*, *ycxD*, and *xy02*, and 168 lacks *yx01*, *aat*, and *xy02*. The Fengycin cluster in DSM7 lacks *fenB*, *fenC*, and *fenD*.

### Genetic basis for the anti-pathogen activity of HNA3.

Genetic comparison of HNA3 genome against the MIBiG database for the detection of secondary metabolites gene clusters shows high sequence homology to the known biosynthetic gene clusters. Twelve active compounds were detected in the HNA3 genome showing 100% of gene similarity against the MIBiG database, two compounds showed more than 80% gene similarity, and 35 active compounds showed between 4% to 45% gene similarity. HNA3 genome encoded a variety of active compounds such as antibacterial, antifungal, and anti-cyanobacterial. In Table 4, We will discuss active compounds that showed more than 80% gene similarity against MIBiG database. Bacillaene (BGC0001089), Difficidin (BGC0000176), and Amylocyclicin (BGC000040) are an antibacterial compound encoded by (6,10, and 11) gene cluster respectively.

**TABLE 4:** Genes and gene clusters of predicated active metabolites detected in the genome of Bacillus velezensis HNA3

Genes and gene clusters or location (Locus tag)	Metabolites	Biological function	Effect against	% of gene cluster similarity against MIBiG database	Reference
*baeB, baeC, baeD, baeE, baeK, baeG, baeH, baeL, baeJ, baeM, baeN, baeR, baeS*	Bacillaene	Antibiotic, Induction of ISR	Bacteria	100%	(55)
*difA, difB, difC, difD, difE, difF, difG, difH, difI, difJ, difK, difL, difM, difN, difO*	Difficidin	Antibiotic	Bacteria	100%	(61)
3,044,009 - 3,044,230 (FHJ82_15040) 3,044,247 - 3,044,774 (FHJ82_15045) 3,044,771 - 3,045,475 (FHJ82_15050) 3,045,468 - 3,047,123 (FHJ82_15055) 3,047,206 - 3,047,541 (FHJ82_15060) 3,047,608 - 3,048,180 (FHJ82_15065)	Amylocyclicin	Antibiotic, Induction of ISR	Bacteria	100%	(62)
*fenB, fenA, fenD, fenC*	Fengycin	Antibiotic, Induction of ISR	Fungi	100%	(56)
*srfA, srfB, srfC*	Surfactin	antibiotics, Induction of ISR	Fungi	82%	(105)
*mycA, mycB, mycC*	Mycosubtilin	Antibiotics, Induction of ISR	Fungi	100%	(58)
*bmyA, bmyB, bmyC, bmyD*	Bacillomycin D	Antibiotics, Induction of ISR	Fungi	100%	(8)
1,885,602 - 1,893,452 (FHJ82_09330) 1,893,541 - 1,909,626 (FHJ82_09335) 1,909,671 - 1,921,619 (FHJ82_09340) 1,921,639 - 1,922,841 (FHJ82_09345)	Paenilarvins	Antibiotic	Fungi	100%	(59)
*ituC, ituB, ituA, ituD*	Iturin	Antibiotics	Fungi	88%	(64)
*pssE, pssD, pssC, pssB, pssA*	Plipastatin	Antibiotics	Bacteria, Fungi	100%	(57)
*bacG, bacF, bacE, bacA*	Bacilysin	Antibiotic	Bacteria, Fungi	100%	(63)
*pks2, pksA, pksB, pksC, pksD, pksE, pksF, pksG, pksH, pksI*	Macrolactin	Antibiotics, Induction of ISR	Bacteria, Fungi	100%	(40)
*paeG, paeA, paeC, paeE, paeB, paeF*	Paenibactin	Siderophore during iron deficiency in soil	Microbial competitors	100%	(65)
*dhbF, dhbB, dhbE, dhbA*	Bacillibactin	Siderophore during iron deficiency in soil	Microbial competitors	100%	(65)

In addition, HNA3 possesses gene clusters (1, 7) involved in the production of some antifungal compounds such as Surfactin (BGC0000433), Fengycin (BGC0001095), iturin (BGC0001098), Bacillomycin D (BGC0001090), Mycosubtilin (BGC0001103), and Paenilarvins (BGC0000402).

Some active compounds detected in clusters (5, 7, 12) have a dual antibacterial-antifungal activity such as Macrolactin (BGC0000181) and Pipastatin (BGC0000407), while Bacilysin (BGC0001184) is a bactericidal and anti-cyanobacterial compound.

It is also notable that HNA3 has gene cluster (11) encoded for two siderophores products (Bacillibactin [BGC0000309], and Paenibactin [BGC000040]), which impedes the growth of bacterial and fungal competitors of phytopathogens by competing for the necessary iron ions and thus plays a significant role in expediting the procurement of ferric ions (Fe^3+^) from minerals and rhizosphere organic compounds (32, 33).

HNA3 genome had 77 genes encoding for possible antifungal and antibacterial CAZymes, such as chitinase (GH18), chitosanase (GH 46), endoglucanase (GH 5,11,16,26,51), lysozyme (GH23,73), and peptidoglycan β-N-acetylmuramidase (GH171), which have the potential to inhibit plant pathogen. Most CAZyme proteins predicated in the HNA3 genome (31%) have amino-terminal with a signal peptide that allows the enzyme to be exported across the membrane, indicating that they are secreting enzymes.

### Genetic basis for the plant growth-promoting activity of HNA3.

HNA3 genome has multiple genes encoding proteins predicted to be associated with plant growth promotion activity that are were illustrated in Table 5, including 11 putative genes involved in the production of indole-3-acetic acid (IAA), Furthermore, genes encoded putative cytochrome P450 synthase and spermidine acetyltransferase, which are predicated to produce spermidine and polyamine. There were also other genes encoding proteins involved in the production of the volatile compound (VOC) 3-hydroxy-2-butanone, such as acetolactate decarboxylase (*alsD*), acetolactate synthase (*alsS*), a transcriptional regulator (*alsR*), and 2,3-butanediol dehydrogenase (*bdhA*). HNA3 genome possesses 13 genes involved in the process of biofilm development and regulation. HNA3 has 19 phosphatase genes involved in phosphorus solubilization, including a *phytase*gene HNA3:2090869:2092020 (Table 6).

**TABLE 5:** Genes detected in Bacillus velezensis HNA3 genome predicated to be involved in plant growth-promoting activity

Gene ID	Gene name	Protein coded by the gene	Reference
Genes detected in Bacillus velezensis HNA3 genome predicated to involved in the production of indole acetic acid (IAA)			
HNA3_GM002195	*trpA*	Tryptophan synthase alpha chain	(70)
HNA3_GM002196	*trpB*	Tryptophan synthase beta chain	(70)
HNA3_GM002198	*trpC*	Indole-3-glycerol phosphate synthase	(106)
HNA3_GM002199	*trpD*	Anthranilate phosphoribosyl transferase	(106)
HNA3_GM002200	*trpE*	Anthranilate synthase component I	(37)
HNA3_GM002197	*trpF*	Phosphoribosyl anthranilate isomerase	(71)
HNA3_GM002011	*dhaS*	Putative aldehyde dehydrogenase	(107)
HNA3_GM003778	*ysnE*	N-acetyltransferase	(107)
HNA3_GM003661	*ywkB*	Auxin efflux carrier	(72)
HNA3_GM002076	*phytase*	Mineralize organic phosphorus enzyme	(107)
HNA3_GM000940	*yhcX*	Putative amidohydrolase	(70)
Genes detected in Bacillus velezensis HNA3 genome predicated to involved in the production spermidine and polyamine			
HNA3_GM001791	*pksS*	Polyketide biosynthesis cytochrome P450	(108)
HNA3_GM003698	*speE*	Spermidine synthase, polyamine metabolism	(76)
HNA3_GM003859	*msmX*	ABC-type spermidine transport systems	(75)
HNA3_GM000582	*speG*	Spermidine acetyltransferas	(76)
Genes detected in Bacillus velezensis HNA3 genome predicated to involved in the production volatile compound (VOC)			
HNA3_GM003543	*alsD*	Acetolactate decarboxylase	(109)
HNA3_GM003544	*ilvB*	Acetolactate synthase	(109)
HNA3_GM003545	*alsR*	Transcriptional regulator	(110)
HNA3_GM000650	*bdhA*	2,3-butanediol dehydrogenase	(110)
HNA3_GM002880	*acuC*	Acetoin dehydrogenase	(45)
Genes detected in Bacillus velezensis HNA3 genome predicated to involved in biofilm formation, development, and regulation			
HNA3_GM001560	*ylbF*	Controls biofilm development	(111)
HNA3_GM001771	*ymcA*	Biofilm development	(9)
HNA3_GM002999	*iolU*	Scyllo-inositol 2-dehydrogenase (NADP(+) involved in biofilm formation protein	(112)
HNA3_GM003376	*slrR*	Master regulator for biofilm formation	(111)
HNA3_GM000103	*cysE*	Serine O-acetyltransferase protein for Microbial metabolism in diverse environments Biofilm formation	(79)
HNA3_GM001715	*fliA*	RNA polymerase sigma factor for flagellar operon and Biofilm formation	(113)
HNA3_GM001805	*hfq*	Host factor-I protein for Quorum sensing Biofilm formation	(9)
HNA3_GM002152	*crr*	Sugar-specific IIA component for Biofilm formation	(78)
HNA3_GM002200	*trpE*	Anthranilate synthase component I for Quorum sensing Biofilm formation	(78)
HNA3_GM002428	*sinR*	Master regulator for biofilm formation	(79)
HNA3_GM002965	*luxS*	S-ribosyl homocysteine lyase for Quorum sensing Biofilm formation	(107)
HNA3_GM003359	*rpoN*	RNA polymerase sigma-54 factor for Biofilm formation	(114)
HNA3_GM003477	*csrA*	Carbon storage regulator for Biofilm formation	(80)
HNA3_GM003483	*flgM*	Negative regulator of flagellin synthesis *flgm*for Biofilm formation	(115)
HNA3_GM003506	*wecB*	UDP-N-acetylglucosamine 2-epimerase for Biofilm formation	(116)
HNA3_GM003513	*tagA, tarA*	Acetylglucosaminyl diphosphoundecaprenol N-acetyl-beta-D-mannosaminyl transferase for Biofilm formation	(117)

**TABLE 6:** Phosphatase genes detected in Bacillus velezensis HNA3 genome predicated to be involved in phosphorus solubilization

Gene ID	Gene name	Reference
HNA3_GM000269	Alkaline phosphatase D *phoD*	(118)
HNA3_GM000410	Phosphoglycolate phosphatase *ycsE*	(118)
HNA3_GM000496	Phosphoserine phosphatase *rsbU*	(119)
HNA3_GM000500	Phosphoserine phosphatase *rsbX*	(120)
HNA3_GM000770	Acylphosphatase	(121)
HNA3_GM000799	Tyrosine-phosphatase *yfkJ*	(120)
HNA3_GM000939	Putative phosphatase *yhcW*	(122)
HNA3_GM000957	Alkaline phosphatase 4	(67)
HNA3_GM001053	Histidine phosphatase *phoE*	(13)
HNA3_GM001168	Putative triphosphatase *yjbK*	(123)
HNA3_GM001173	Bis(5′-nucleosyl)-tetraphosphatase *prpE*	(13)
HNA3_GM001529	Inositol-1-monophosphatase	(124)
HNA3_GM001642	Protein phosphatase *prpC*	(81)
HNA3_GM002247	Alkaline phosphatase synthesis transcriptional regulatory protein *resD*	(118)
HNA3_GM002535	HAD family phosphatase *yqeG*	(119)
HNA3_GM002574	Uracil phosphatase *ybjI*	(125)
HNA3_GM002794	Alkaline phosphatase synthesis sensor protein	(118)
HNA3_GM002795	Alkaline phosphatase synthesis transcriptional regulatory protein *phoP*	(118)
HNA3_GM002076	*phytase*	(107)

### Genetic basis for the participation of HNA3 in symbiotic N2 fixation and nodulation.

HNA3 has genes (QJC41574, QJC43511, and QJC42170) that are predicted to produce arabinogalactanase, pectate lyase, and xylanase enzyme, and also has *guaB* gene that is predicted to participate in the nodulation process if HNA3 co-inoculated with rhizobium. Three *nif* genes (*nifU*, *nifS*, and *nifF*) have been detected in the HNA3 genome that are predicted to be involved in the synthesis of cysteine and flavodoxin biosynthesis.

Interestingly, the HNA3 genome has the *htrA* and *htpG* genes, which are involved in tolerance to high temperature, and also has the *cspB* gene that encodes cold shock proteins (Csps).

## DISCUSSION

The world is faced with the high necessity of developing sustainable and eco-friendly methods to improve agricultural productivity (34). Great efforts have been made to discover new PGPR and develop PGPR inoculum for the development of highly efficient biofertilizers and biofungicides to substitute existing health threatening methods. Genomic studies of new isolates can explain the biological mechanism, as well as help to discover new biological activities (35). In 2013, the HNA3 strain was isolated by our group. Xu et al. demonstrated the broad spectrum antifungal activity of HNA3 extract and highlighted the antifungal activities of the lipopeptide metabolites secreted by HNA3 (27).

In this study, the whole genome of HNA3 was sequenced and comparative analysis was performed to identify its phylogenetic position and investigate genes involved in plant growth promotion and biocontrol effects. We investigated the evolutionary relationships of HNA3 with previously sequenced 74 *Bacillus* strains (seven B. paralicheniformis strains, 21 B. subtilis strains, 18 B. amyloliquefaciens strains, and 28 B. velezensis strains) to determine the HNA3 species at the taxonomic level. Our collection was chosen based on two facts. First, B. velezensis HNA3 was identified previously as B. amyloliquefaciens based on 16S rRNA sequence analysis (27). Secondly, B. subtilis, B. amyloliquefaciens, and B. velezensis species were recognized, yet for many years these species have been difficult to identify using standard phenotypic techniques (36). Furthermore, due to the highly conserved character of the genes, phylogenetic analysis of the 16S rRNA gene fails to distinguish species within the complex (37). The phylogenetic trees created based on 293 single copy core genes shared by all strains showed that HNA3 strain together with B. amyloliquefaciens strains and B. velezensis strains formed an independent branch in the phylogenetic tree. Then HNA3 formed a clade with B. velezensis DR-08, CBMB205, LDO2, JT3-1, ZF2, sx01604, GQJK49, and JTYP2 with the same node, which was also supported by the ANI analysis results.

Previously, B. subtilis and B. licheniformis were identified as the “original members” of the genus *Bacillus* (38) and each of them forms a branch in the phylogenetic tree with distinctive genetic properties (39), while B. amyloliquefaciens and B. velezensis emerged from B. subtilis as distinct species (40), which is highly consistent with our phylogenetic tree result that HNA3 was classified into the same branch together with B. amyloliquefaciens and B. velezensis strains. The phylogenetic tree among HNA3 and the 15 most closely related strains verify that it is the property of being a *velezensis* species because they shared a similar genomic size range and number of CDSs. At the nucleotide level HNA3 genome exhibited > 98% sequence similarity with the genome of 15 closely related strains and > 99.1% with those of B. velezensis JT3, LDO2, and CBMB205, which is consistent with the fact that all of these strains were found in the same cluster in the phylogenetic tree. Based on the whole genomic analysis of HNA3, we shall correct the results published in 2013. Xu et al. classified HNA3 as B. amyloliquefaciens based on morphological observations and 16S rRNA sequence analysis, but the results of the present study clearly demonstrate that HNA3 belongs to B. velezensis (27).

We further used the COG assignment to ascertain the functional categories of the protein coding genes of HNA3 and core genome of 15 closely related strains. The results showed that HNA3 comprises a larger proportion of genes involved in amino acid, carbohydrate transportation, and metabolism (categories G and E), and secondary metabolite biosynthesis gene clusters. The diversity of carbohydrate and amino acid metabolism genes besides secondary metabolite biosynthesis in the bacterial genome improves the genetic adaptation to the various nutrient environment in the field application (41, 42).

*Bacillus* is a large and heterogeneous bacterial genus with wide environmental distributions and variations (43). The genetic content of the bacteria changes, so it can perform its task to the fullest (44). Because B. subtilis, B. amyloliquefaciens, and B. velezensis are very similar species in phenotype and genotype (25), there are differences in traits that change depending on the biological environment of each species which led to their splitting in the phylogenetic tree (45). *Bacillus*168 contained the highest genetic content, followed by HNA3, FZB42, and DSM7. The genome size and GC content of microorganisms impact their comprehensive environmental adaptability under different conditions (46). Strains with larger genome sizes are usually of higher adaptability to various conditions because they can encode additional products for metabolism and stress tolerance (47). Numerical comparison of CDSs among the four *Bacillus* strains revealed that HNA3 shared 3,505 genes with B. velezensis FZB42 and 3,358 genes with B. amyloliquefaciens DSM7 which indicates that HNA3 has a large, shared number of genes with the B. velezensis species level (48). Besides, HNA3 and FZB42 have the same performance as PGBR.

CAZymes comparison showed that one predicated protein feruloyl esterase has been found only in the genome of HNA3 that catalyzes the hydrolysis of arabinose. Without a doubt, carbohydrate active enzymes play a crucial role in the genome of any bacteria, especially *Bacillu*s species (18). It assists the breakdown of complex compounds into simpler substances that are easier to process and absorb (49), implying that their presence and changes in the genetic content of bacteria are the opportunity to survive in different environments and utilize them as a source of nourishment (50). It is also important in the eradication of plant diseases by disintegrating the pathogen’s cell wall (51). The existence of carbohydrate active enzymes in the genetic material of B. velezensis strains is significant in numerous ways, including the strain’s capacity to suppress the pathogen and its absorption of various nutrients (48). Enzymes of interest can also be isolated and used industrially (52).

Based on comparative genomics investigation, one gene cluster (4) expressing Lanthipeptide was discovered solely in B. velezensis HNA3 by genome mining using anti-SMASH software version 5.1.0 and may lead to the production of novel bioactive substances. The analysis of gene clusters indicated that rich gene clusters are distributed among B. velezensis strains, and certain gene clusters are conserved across strains. This study demonstrates that B. velezensis strain HNA3 and other B. velezensis strains have the potential to be employed as PGPR and biocontrol agents (53).

Currently, various strains of B. velezensis, a typically PGPR, have received considerable attention by developing various forms of secondary and biologically active metabolites to regulate a wide range of soilborne diseases and stimulate plant growth (54). Twelve gene clusters have been identified in the genome of B. velezensis HNA3 responsible for the synthesis of 14 secondary metabolites identified by the comparative genomic analysis, including, Bacillaene (55), Fengycin (56), Bacillomycin D (8), Surfactin (57), Plipastatin (57), Mycosubtilin (58), Paenilarvins (59), Macrolactin (60), Difficidin (61)Amylocyclicin (62), Bacilysin (63), Iturin (64), Bacillibactin (65), Paenibactin (65), and others (unknown).The PGPR distinguishing feature is its ability to produce various antimicrobial compounds that enable it to be free-living in soil (66). Based on the gene cluster analysis, HNA3 is predicted to produce active strong secondary metabolites with antibacterial, antifungal, and anticyanobacterial activities, which gives it a significant advantage due to the ability of these compounds to inhibit plant pathogens (67). Besides producing antimicrobial metabolites, the HNA3 genome had 77 genes encoding for possible anti-fungal- and anti-bacterial-secreting carbohydrate active enzymes. It has been reported that the ability of hydrolysis-active enzymes to penetrate fungal wall leads to the rapture of fungal cell and finally killed (68). Because the genome of B. velezensis strains is known to harbor hydrolysis-active enzymes, this increases its importance and effectiveness as biocontrol inoculum (69).

Some putative genes related to plant growth-prompting activity have been discovered in the HNA3 genome. HNA3 genome has genes such as *trpA*, *trpB*, *trpC*, *trpD*, *trpE*, and *trpF* that participate in the synthesizing of IAA from indole-3-acetonitrile (70, 71). Furthermore, the gene *ysnE* is thought to encode an IAA transacetylase implicated in the tryptophan-independent IAA biosynthesis pathway (72). As a result, we believe that B. velezensis HNA3 contains several genes that are functionally linked to auxin synthesis and play important roles in its capacity to stimulate plant development (73). Also, *pksS*, is gene-encoded putative cytochrome P450, while *speE*, *msmX*, and *speG* are gene-encoded spermidine and polyamine synthesis that are suggested to have a role in plant development and growth promotion by involving in the production of active metabolites such as steroids, vitamin D3, cholesterol, cytokinin, statins, and terpenes (74). Aside from the production of spermidine, which is essential for plant development under stress conditions, biofilm production and plant systemic susceptibility to pathogens is also important (75, 76). Besides, *alsD*, *ilvB*, *alsR*, *bdhA*, and *acuC* genes have been detected to encode acetoin, one of the active bacterial volatile compounds released to stimulate the induced systemic resistance (ISR) of plants (77).

The purpose of biofilm, which is made up of polysaccharides, proteins, nucleic acids, and lipids, is to increase cell stability, adhesion, cohesion, interconnection, and temporary immobilization (78). The development of biofilms in plant rhizospheres can enhance the growth of plants and defend them against infectious microbes through systemic resistance and the secretion of antimicrobial compounds (79). HNA3 possesses multiple genes involved in biofilm development; as it is reported, numerous genes are involved in the synthesis of Fengycin, Bacillibactin, and Bacilysin upregulated during biofilm formation (80). HNA3 has 19 phosphatase genes involved in phosphorus solubilization, including a *phytase*. Stimulating plant development rhizobacteria aids plant growth directly by increasing the resource acquisition, one of which is phosphorus (13) that increases soil fertility and, hence, increases crop yield (81).

As it is reported in different researches, some B. velezensis strains improve the nodulation process if co-inoculated with rhizobium. We expect that HNA3 may improve nodulation because some genes related to nodulation are detected in the HNA3 genome such as QJC41574, QJC43511, and QJC42170 genes, and these genes are predicted to produce arabinogalactanase, pectate lyase, and xylanase enzyme, which is suggested to weaken the cell wall of root hairs to facilitate the bacterial invasion for nodulation (82, 83). Furthermore, previous studies have shown that the *guaB* gene, which encodes guanine, is expressed in the early stages of nodule formation (84, 85). *Bacillus* strains’ capacity to produce spores is one of their most significant features, as it enhances their resilience to a range of stress conditions (86). This enables their usage as PGPB inoculants in agricultural and bioremediation processes (66). HNA3 genome harbor putative genes involved in tolerance to severe environmental conditions such as *htrA*, *htpG*, and *cspB* which are involved in tolerance to high temperature and cold shock conditions. Such strains can survive in wide various environments (87).

### Conclusion.

HNA3 is a prospective PGPR. The analysis of its genome and genetic comparison demonstrated that it is a B. velezensis strain with different genetic traits from analogs in various biological environments. HNA3 has excellent PGPR potential and directs most genes to consume different sources of substrates to be adapted to different environments. It also possesses the most important characteristics of PGPR such as producing antifungal and antibacterial antibiotics and promoting plant growth. HNA3 has the capability to form endospores that can survive in difficult environmental conditions. Besides, HNA3 belongs to the genus of *Bacillus*, which increases its advantages in agriculture applications. Dry powders can also be produced with a long shelf life. Thus, HNA3 can be formulated and commercially prepared for field application to suppress phytopathogens and improve crop development, either alone or as part of microbial associations.

## MATERIALS AND METHODS

### Bacterial strains.

B. velezensis HNA3 was isolated from rhizosphere soil (27) and stored in our laboratory (Key Laboratory of Agricultural Microbiology, Huazhong Agricultural University). HNA3’s whole genome was sequenced and submitted to GenBank (accession number CP040881). Our sources for creating core genomic phylogenetic analysis comprised seven B. paralicheniformis strains, 21 B. subtilis strains, 18 B. amyloliquefaciens strains, and 28 B. velezensis. The NCBI GenBank server was used to download the genome of all strains.

### Genome sequencing and assembly.

A single colony of the stored HNA3 strain was swiped onto an LB agar plate and cultivated overnight at 28°C, followed by one colony transferred to a 50-mL LB medium and cultured overnight. The SDS technique was used to extract genomic DNA (88). DNA was extracted using a standard procedure that comprised mechanical segmentation followed by enzymatic lysis and (a 10%-SDS solution was added to bacterial broth culture to a final concentration of 1% with 4 μL of proteinase K solution). DNA was extracted and washed with 80% ethanol. The extracted DNA was detected and quantified using agarose gel electrophoresis and Qubit.

HNA3 genome was sequenced using SMRT technology. The readings were assembled using the SMRT Link v5.0.1 software. After obtaining the first assembly results, the distribution of sequencing depth was recorded and the preliminarily assembled sequence was identified as a chromosome based on its length and alignment technique. The SMRT Link v5.0.1 was used to filter the low-quality reads, and the filtered reads were combined to create a single contig with no gaps. Sequencing was performed at the LC-BioTechnology Co. Ltd., Hang Zhou, Zhejiang Province, China (89).

### Phylogenetic analysis.

By analyzing the subset of SNPs identified in all single-copy core genes across genomes, the evolutionary connections between HNA3 and the fully sequenced *Bacillus* were inferred. The SNPs were introduced in the order of the HNA3 genome’s single-copy genes. By extracting the collection of SNPs, we were able to identify potential crossover regions (90). Multiple sequence alignment was carried out with the MAFFT alignment tool (https://www.ebi.ac.uk/Tools/msa/mafft/) and BMGE filter MSA. The evolutionary distance was calculated using the maximum likelihood method with 100 bootstrap replicates using an IQ tree (http://www.iqtree.org) (91). The phylogenetic tree was generated using the interactive tree of life iTOL (https://itol.embl.de) software.

### Analysis of average nucleotide identity.

The ANI was used to determine the genomic diversity. This method can delineate species to indicate diversity at the genomic level. The genomes of sequenced *Bacillus* strains were compared with the B. velezensis HNA3 genome using the average nucleotide identity analysis tool FastANI (92).

### Gene functional category.

To predict coding DNA sequences of the HNA3 genome, Glimmer 3.02 was utilized (93). Then the alignment of HNA3 CDSs against many commonly used databases was performed including the NCBI non-redundant (NR) database, Swiss-Prot, COGs, gene ontology (GO), and KEGG (Kyoto encyclopedia of genes and genomes). The functional classification of the genes of HNA3 and the core genome of 15 closely related strains were performed using COG assignment (94).

### Comparative genomics.

For the comparison between the genome of B. velezensis HNA3 and other bacillus strains, each member of the *Bacillus* genome was aligned by using the BRIG with the reference genome of B. velezensis HNA3 (95). The Venn diagram was used to compare the CDS of HNA3 with that of FZB42, DSM7, and 168.

WebACT (96) was used to identify the conserved and specific genome regions responsible for similar biological behaviors or different environmental adaption properties. To compare genetic components like pseudogenes, we used Pseudofinder, which is a Python3 script that detects pseudogene candidates from annotated GenBank files (97). The genomic island was predicted by using the software Zisland_Explorer accessible at the website http://cefg.uestc.edu.cn/Zisland_Explorer/ or http://tubic.tju.edu.cn/Zisland_Explorer/ (98). The prophage was analyzed by using the software phispy at the site of https://sourceforge.net/projects/phispy/ (99). RNAmmer was used to explore rRNA (100). Transporter Classification Database (TCDB) was used to predict the transporter (101).

### Secondary metabolites analysis.

AntiSMASH 5.0 software (102) was used to identify the gene cluster related to secondary metabolites. Using antiSMASH 5.0 on the default settings, the structure of secondary metabolism was detected against MIBiG.

### Carbohydrate active enzyme identification.

Putative genes encoding for the CAZymes, such as GH, PL, CE, GT, AA, and CBM families were discovered in the HNA3 genome using the dbCAN2 (http://bcb.unl.edu/dbCAN2/) database, which included the dbCAN CAZymes domain (by HMMER search), short, conserved motifs (by Hotpep search), and CAZy databases (by DIAMOND search) (103). The putative CAZyme genes were then analyzed using the SignalP 5.0 software to seek signal peptides (104).

### Data availability.

The gene sequence of HNA3 is available at the NCBI website with accession No. CP040881, the URL of the gene sequence is: https://www.ncbi.nlm.nih.gov/nuccore/CP040881.1/.
