# Supplementary material for: Comparative Genome Analysis Reveals Phylogenetic Identity of Bacillus velezensis HNA3 and Genomic Insights into Its Plant Growth Promotion and Biocontrol Effects
Source: Microbiol Spectr. 2022 Feb 2;10(1):e02169-21. doi: 10.1128/spectrum.02169-21 (PMC8809340; doi:10.1128/spectrum.02169-21)
Supplement: SUPPLEMENTAL FILE 1 — Supplemental material. Download SPECTRUM02169-21_Supp_1_seq9.pdf, PDF file, 0.5 MB [file spectrum02169-21_supp_1_seq9.pdf]

Supplementary Fig. S1

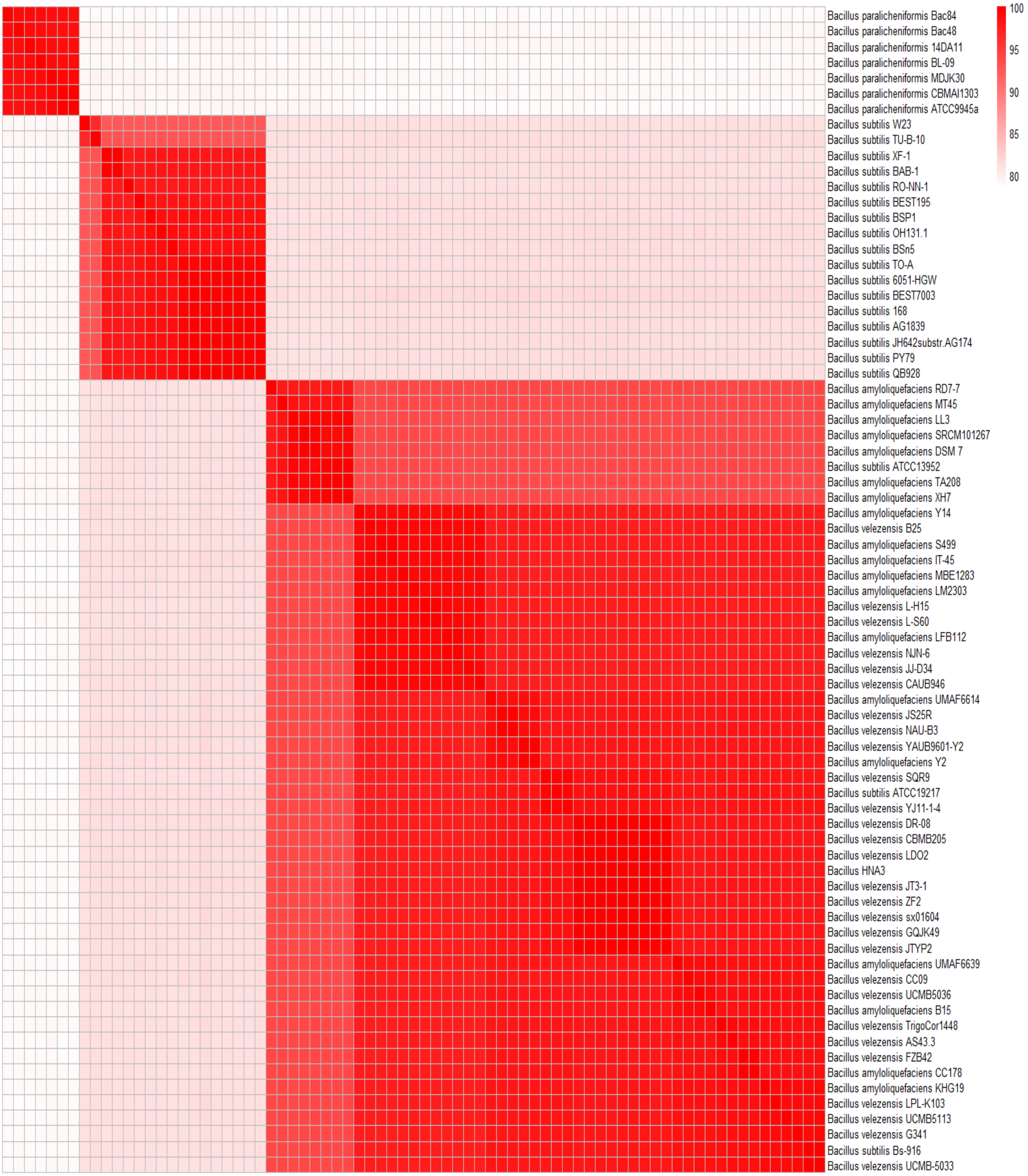

Supplementary figure S1. ANI analysis for 74 *Bacillus* strains. Red color indicates high percentage of similarity. Dark red color indicates high value of ANI. Light red color indicates low value of ANI.

Supplementary Fig. S2

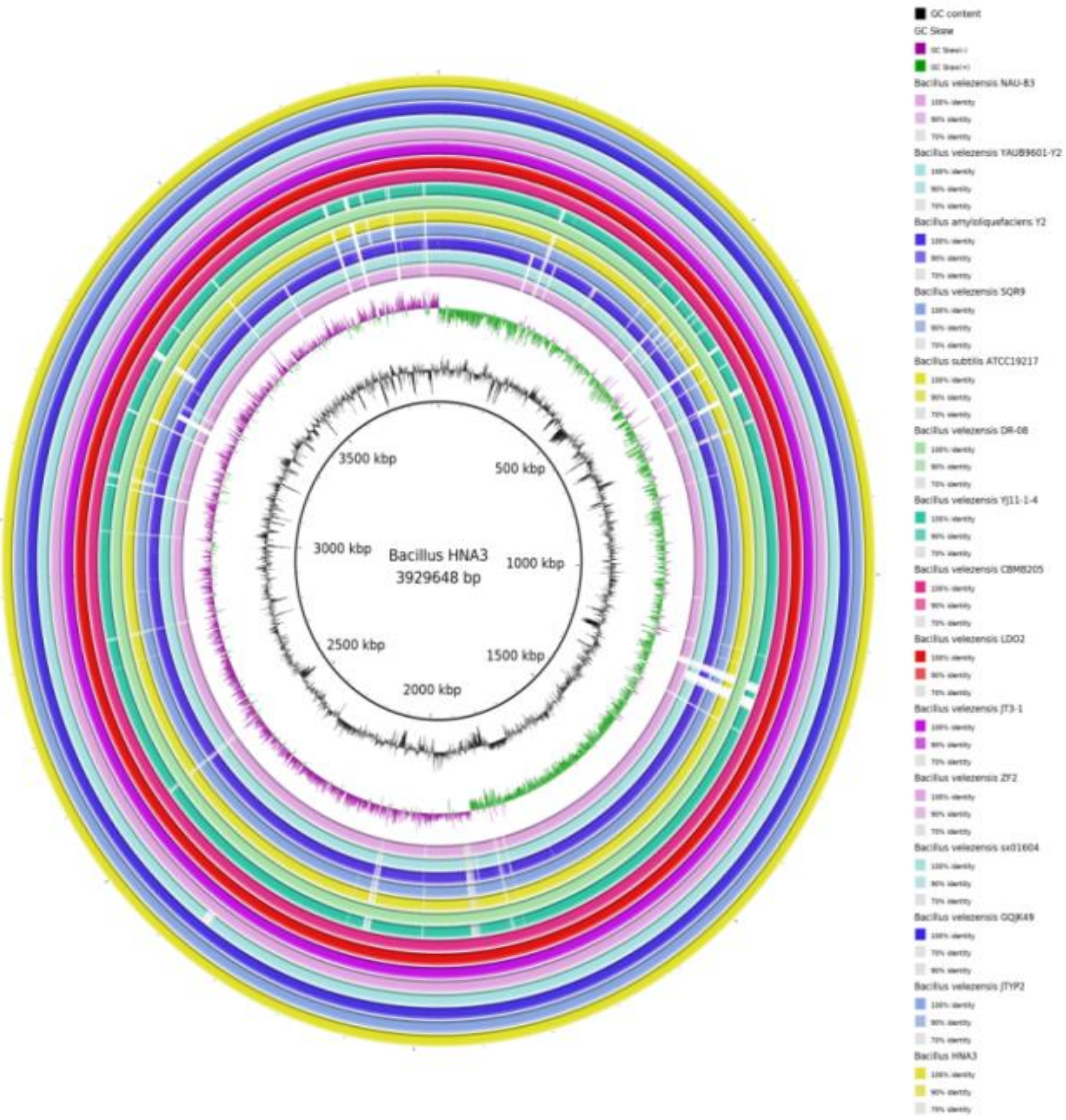

Supplementary figure S2. Sequence alignment between HNA3 and fifteen closely related strains by using BLAST Ring Image Generator (BRIG)

Supplementary Fig. S3

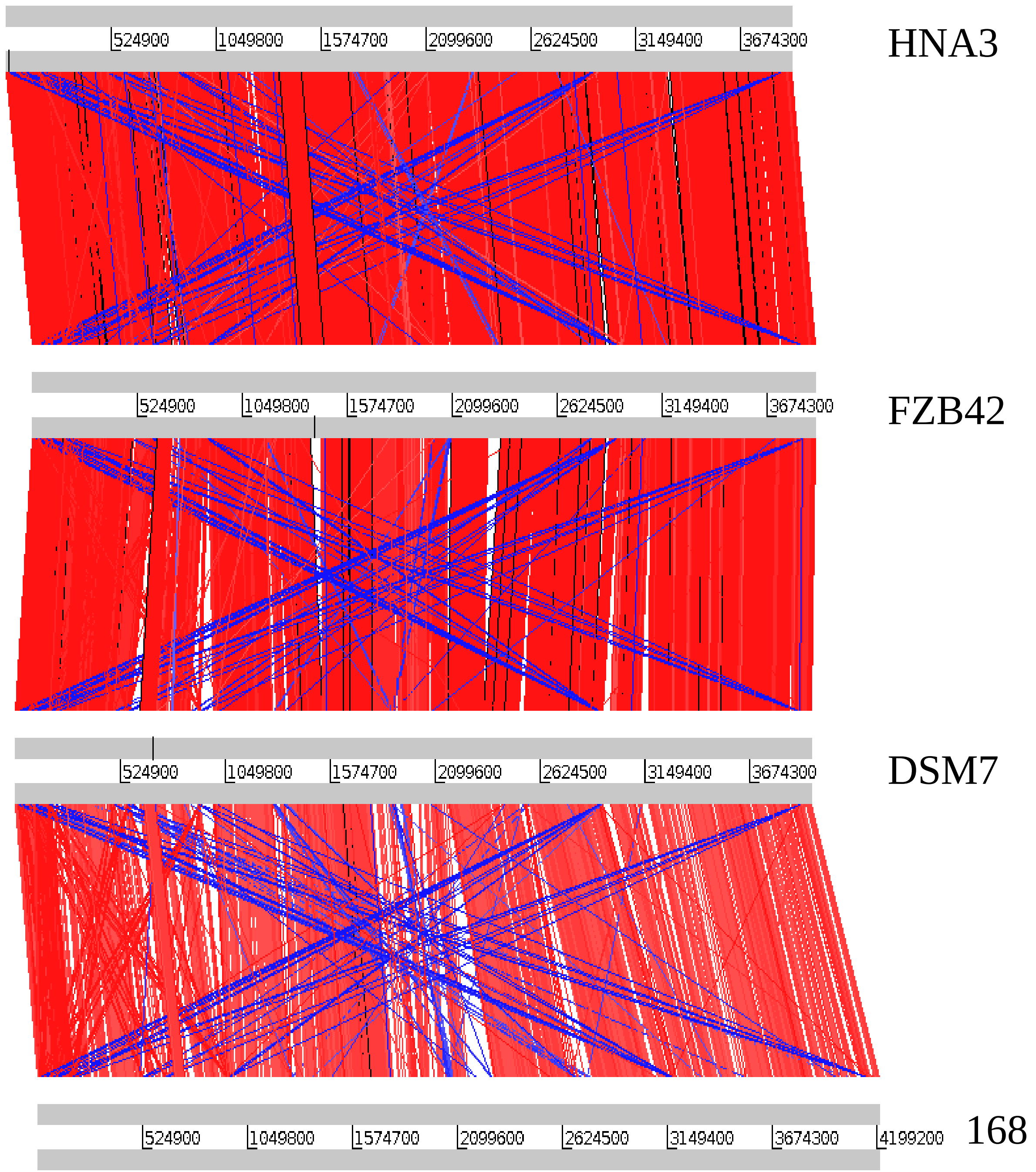

Supplementary figure S3. Chromosomal structures of four genomes. Red color indicates co-linear regions, and blue color indicates inverted regions. From top to bottom is *Bacillus* HNA3, *Bacillus velezensis* FZB42, *Bacillus amyloliquefaciens* DSM7 and *Bacillus subtilis* 168.
